# Challenges in Diagnosis and Management of Coffin–Lowry Syndrome—Single-Center Experience

**DOI:** 10.3390/diagnostics16070990

**Published:** 2026-03-25

**Authors:** Ana Maria Chirilas, Alexandru Cărămizaru, Anca-Lelia Riza, Andreea Mitut-Veliscu, Andrei Costache, Rebecca-Cristiana Șerban, Aritina Morosanu, Carmen Niculescu, Alexandru-Cătălin Pâslaru, Florin Burada, Ioana Streata

**Affiliations:** 1 Regional Centre of Medical Genetics Dolj, Emergency County Hospital Craiova, 200642 Craiova, Romania; anamariachirilas@gmail.com (A.M.C.); andreea.crgm@gmail.com (A.M.-V.); rebecca.serban@umfcv.ro (R.-C.Ș.); florin.burada@umfcv.ro (F.B.); ioana.streata@umfcv.ro (I.S.); 2 Laboratory of Human Genomics, University of Medicine and Pharmacy of Craiova, 200638 Craiova, Romania; 3 Doctoral School, University of Medicine and Pharmacy of Craiova, 200349 Craiova, Romania; 4 Department of Biophysics, University of Medicine and Pharmacy of Craiova, 200638 Craiova, Romania; 5 Department of Pediatrics, University of Medicine and Pharmacy of Craiova, 200638 Craiova, Romania; aritina.morosanu@umfcv.ro (A.M.); carmen.niculescu@umfcv.ro (C.N.); 6 Faculty of Medicine, “Carol Davila” University of Medicine and Pharmacy, 050474 Bucharest, Romania; catalin.paslaru@umfcd.ro

**Keywords:** Coffin–Lowry syndrome, syndromic intellectual disability, *RPS6KA3* gene

## Abstract

**Background/Objectives**: Coffin–Lowry syndrome (CLS) is a rare X-linked disease caused by pathogenic variants in the *RPS6KA3* gene. It is generally characterized by syndromic intellectual disability and distinctive facial features, skeletal abnormalities, stimulus-induced drop attacks in males, and variable manifestations in females. **Methods**: We report clinical and genetic findings in a series of 10 cases, eight males and two females, evaluated at the Regional Centre of Medical Genetics Dolj—Emergency Clinical County Hospital Craiova. **Results**: Genetic testing identified 10 *de novo* variants in the *RPS6KA3* gene consisting of six missense mutations, one nonsense variant, one frameshift, and two variants in non-coding or intronic regions. Case management requires multidisciplinary coordination and is limited to resources mostly available in reference centers. **Conclusions**: CLS highlights the importance of molecular diagnosis in rare genetic disorders, particularly when clinical features are subtle or atypical. These findings have practical implications for clinical management, suggesting the need for comprehensive genetic screening and individualized care approaches.

## 1. Introduction

Coffin–Lowry syndrome (CLS) OMIM #303600 is a rare X-linked dominant neurodegenerative disorder [1]. The estimated incidence of CLS ranges between 1 in 50,000 and 1 in 100,000 individuals [2], although some authors suggest a higher frequency of 1 in 40,000 to 50,000 based on clinical experience [3]. To date, no extensive epidemiological studies have been published.

The genetic disorder was first described by Coffin and Lowry in individuals who shared similar clinical and phenotypic features. Hanauer et al. clarified the pattern of inheritance, and by 1996, the causative gene was identified [1,2,4]. Approximately 70–80% of CLS cases are considered sporadic, with *de novo* genetic variants responsible for around two-thirds of cases [2,4].

The associated gene, ribosomal protein S6 kinase polypeptide 3 (*RPS6KA3*), Xp22.12, encodes a member of the RSK (ribosomal S6 kinase) family of growth factor-regulated serine/threonine kinases. Its coding region has 22 exons and encodes a 740-amino-acid protein; more than 150 variants of the gene have been linked to CLS [5,6,7], including variants of unknown significance. RSK2 has important roles in cellular proliferation, differentiation, and survival but also plays a key role in brain function. Animal models in mice reproduce the neurological deficits seen in CLS deficits [8]—in RSK2-deficient mice, hippocampal ERK1/2 activity is increased, suggesting that the loss of RSK2-mediated negative feedback on ERK1/2 contributes to brain dysfunction. RSK1 and RSK2 are expressed in brain regions involved in learning and intelligence, with RSK activity enriched at synapses. The experimental inhibition of RSK impairs long-term memory [7,8,9,10].

CLS is characterized by developmental delay, intellectual disability, neurologic manifestations, musculoskeletal manifestations, and dysmorphic features involving the face and hands [3,11,12,13]; as such, the disease is also referred to as *RPS6KA3*-related intellectual disability. CLS occurs mostly in males. Male phenotypes are variable and include nonspecific neurologic findings, as well as stimulus-induced drop episodes. Together with tapered fingers and the characteristic facial profile, these features are indicative of Coffin–Lowry syndrome, which can be confirmed through molecular genetic testing [14,15,16,17,18]. Establishing the diagnosis is more challenging in females, as they present milder phenotypes, in some cases represented by non-syndromic mild intellectual disability.

Having key clinical features of Coffin–Lowry syndrome does not make diagnosis less difficult. In very young patients, the phenotypic features are milder and nonspecific compared with those observed later in life [19,20]; there is substantial phenotypic variability and limited awareness among non-specialists. Additionally, the genetic diagnosis is challenging in itself—there is a wide spectrum of *RPS6KA3* variants, many of which are rare or newly identified [21,22,23].

Patients with Coffin–Lowry syndrome require multidisciplinary follow-up. The disorder affects multiple systems, including the nervous and cardiovascular systems [24,25,26,27]. Knowledge of the natural history is currently limited; only few long-term observations exist. Available reports suggest progressive musculoskeletal deterioration and additional complications that reduce quality of life over time [11,28,29]. Current management guidelines remain largely consensus-based and focus on supportive care rather than evidence-driven interventions, reflecting the scarcity of longitudinal and genotype-informed data [11,25,30]. Key unmet needs include clearer genotype–phenotype correlations, a better understanding of factors contributing to diagnostic delay, and regional-specific considerations that may influence access to specialized evaluation [26,31]. Addressing these gaps is essential for improving early recognition, guiding clinical management, and informing future therapeutic strategies.

The aims of the current study are to report clinical and genetic findings, as well as the diagnostic path of 10 Romanian patients with CLS. These patients were referred for comprehensive clinical and genetic evaluation and subsequently underwent genetic testing. Our study adds to the current understanding by offering detailed data on rare genetic variant types, identifying crucial obstacles leading to diagnostic delays, and revealing phenotypic differences that shape the clinical presentation and diagnostic pathway. In our cohort, diagnostic delay was primarily related to the wide variability of clinical manifestations and, in some cases, limited access to timely genetic testing.

## 2. Materials and Methods

### 2.1. Study Design and Setting

This study is a retrospective case series conducted at the Regional Centre of Medical Genetics Dolj (CRGMDj) within the Emergency Clinical County Hospital Craiova (ECCHC-SCJUC), one of the expertise centers for genetic neurodevelopmental disorders in Romania. The study was approved by the institutional ethics committee of ECCHC, and written informed consent was obtained from the parents or legal guardians of all participants.

### 2.2. Patient Selection

We reviewed the medical records of all patients diagnosed with Coffin–Lowry syndrome between January 2020 and December 2025. A total of 10 patients with confirmed molecular diagnoses of CLS were included in the study. The inclusion criteria consisted of the following:

- Clinical features consistent with CLS (intellectual disability, craniofacial dysmorphism, skeletal anomalies, etc.);
- Molecular confirmation of a pathogenic, likely pathogenic, or uncertain significance variant (VUS) in the *RPS6KA3* gene;
- Availability of clinical data.

Patients without genetic confirmation of the diagnosis were excluded.

### 2.3. Clinical Evaluation

Demographic and clinical data were collected from electronic and paper medical records, standardized interviews with caregivers when available, and extraction of diagnostic and neurodevelopmental information using a predefined data-collection form. Collected variables included age at symptom onset; age at molecular confirmation; developmental history; neurological examination findings; behavioral and psychiatric features; and documented skeletal, audiological, or cardiac anomalies. Each patient underwent a comprehensive physical and neurological examination by a multidisciplinary team including a clinical geneticist, cardiologist, pediatric neurologist, and pediatrician.

Clinical diagnoses, symptoms, and signs were mapped, when applicable, to standardized terminologies, including HPO for phenotyping, ICD-10 for medical diagnoses, and DSM-5 criteria for behavioral and psychiatric classifications. Intellectual disability severity relied on developmental milestones and functional abilities were abstracted from clinical notes using a structured checklist to optimize comparability across participants and minimize classification bias when standardized assessments (DSM-5-aligned behavioral evaluations or validated developmental scales) were not available. The skeletal severity scale (for Coffin–Lowry syndrome) we used primarily evaluates spinal deformity, especially kyphoscoliosis. It is typically divided into mild (minimal vertebral body deformity with mild spinal curvature), moderate (vertebral wedging present with progressive kyphosis and/or scoliosis), and severe (significant vertebral body deformities with marked kyphoscoliosis and functional impairment) categories.

### 2.4. Genetic Testing

Depending on the time of referral and availability, testing consisted of extensive next-generation sequencing (NGS) panels or Whole Exome Sequencing (WES). Copy-number analyses such as MLPA, aCGH, or SNP-array were not performed in this cohort. Genetic analysis was performed in our center or in other accredited laboratories—for 5 patients, genetic testing was performed at CRGMDj, while for another 5, genetic confirmation was obtained in an external laboratory. Genetic diagnostic testing in our center was performed using an extensive next-generation sequencing (NGS) panel, Illumina^®^ TruSight™ One, on NextSeq550 IVD, MID-output to reach 100× mean coverage. Library preparation and sequencing were performed according to the manufacturer’s instructions (Illumina, San Diego, CA, USA). The gene list is available at https://www.illumina.com/content/dam/illumina-marketing/documents/products/gene_lists/gene_list_trusight_one.zip (accessed on 1 July 2024). Validation of NGS findings and segregation trio-analysis was performed using Sanger sequencing (BigDye Terminator v.1.1, ABI3500Dx, CA, USA) and custom-made primers. The LINE insertion was validated through long-range PCR and targeted mRNA sequencing.

### 2.5. Data Analysis

Descriptive analysis was used to summarize clinical features, diagnostic timelines, and management interventions. Continuous variables are reported as medians and ranges and categorical data as frequencies and percentages. No inferential statistics were applied due to the small sample size.

All genetic data generated was re-analyzed using the same secondary and tertiary bioinformatic analysis. Paired-end 2 × 150 bp reads were mapped to the human genome reference sequence (GRCh37, iGenomes resource bundle) and pushed through the nf-core/sarek 3.2.1 pipeline. The germline variants identified were annotated using the ENSEMBL variant effect predictor (VEP), with several plugins for predictive scores; online aggregate databases such as OMIM, ClinVar, and Varsome were also consulted. Variants were classified according to ACMG/AMP 2015 guidelines [32]. Confirmatory testing and cascade testing of family members’ data were analyzed using Mutation Surveyor software version 5.0. There is a reasonable possibility that a variant pathogenicity class could be reclassified in the future once supporting cases are published or functional data becomes available.

## 3. Results

### 3.1. Clinical Findings

CLS is often diagnosed based on clinical evidence, radiological findings, and molecular testing. We included 10 patients, eight males and two females, who presented with clinical features suggestive of CLS (Table 1) and underwent genetic testing.

All eight male patients exhibited the characteristic signs of CLS: moderate to severe intellectual disability (100%), global developmental delay (100%), craniofacial dysmorphism (100%), skeletal anomalies (100%)—most commonly, pectus deformities and progressive kyphoscoliosis—and generalized hypotonia (70%). The two female patients showed more variable and attenuated phenotypes, complicating early clinical suspicion of CLS. In one case, the diagnosis was established only after genetic testing.

Follow-up intervals ranged between 6 and 36 months (median: approx. 12 months). Most patients required multidisciplinary and long-term management, frequently involving neurology (80%), orthopedics (70%), physiotherapy (90%), audiology (60%), and developmental pediatrics services (100%). Core clinical information—including developmental history, neurological assessment, skeletal findings, and audiologic evaluation—was available for all individuals. Overall, the clinical course was largely stable across major systemic domains. The only progressive change observed during follow-up concerned scoliosis, with documented radiographic progression in a subset of patients, reinforcing the need for continued orthopedic surveillance. Among patients with epilepsy, antiepileptic treatment resulted in good seizure control, with no reported worsening of seizure frequency or severity during the follow-up period. Treatment adjustments, when required, were associated with favorable clinical response. Importantly, no cardiac abnormalities were identified during follow-up. No additional complications emerged during the follow-up period.

The completeness of longitudinal documentation varied, reflecting differences in referral patterns, timing of diagnosis, and availability of expert evaluations. Skeletal complications were prevalent, with progressive scoliosis requiring bracing or surgical referral in four patients. Sensorineural hearing loss was present in five patients (50%), generally ranging from mild to moderate severity. Management included hearing aids in three patients, while two required only periodic audiologic monitoring.

Behavioral issues—including aggressivity/anxiety (40%) and sleep disturbances (40%)—were managed through behavioral therapy and/or pharmacologic interventions when indicated. The severity of symptoms varied but mostly fell in the mild to moderate range.

Below, we are showcasing one clinical case that illustrates the variable phenotype observed in heterozygous females with Coffin–Lowry syndrome.

#### Clinical Vignette CLS in a Female Patient—Case 2

This female patient was born at term following an uneventful pregnancy and delivery. Early neurological abnormalities were noted, including generalized hypotonia at 5 months of age. Seizures began at 6 months, initially presenting as infantile spasms. Despite treatment, her epilepsy evolved to focal impaired-awareness seizures, with only partial therapeutic control.

Brain magnetic resonance imaging at 12 months demonstrated mild cortical atrophy, ventriculomegaly, and partial hypoplasia of the corpus callosum.

Developmental delays became evident in early childhood. Independent ambulation was achieved at 18 months, and expressive language delay persisted. Formal cognitive evaluation revealed a mild intellectual disability (IQ 65–70).

At 3 years of age, a physical examination showed nystagmus, subtle dysmorphic features, including hypertelorism, down-slanting palpebral fissures, broad nasal tip, and tapering fingers with mild distal phalangeal hypoplasia. Height was −2.3 standard deviations below the mean. Neurological examination confirmed generalized hypotonia without focal deficits. There was no history of stimulus-induced drop episodes. Cardiac evaluation was normal. Spinal radiographs identified mild scoliosis, which was managed conservatively.

Molecular testing performed at approximately 2 years of age identified a heterozygous pathogenic variant in *RPS6KA3*. Parental testing confirmed the variant to be *de novo*, establishing the diagnosis of Coffin–Lowry syndrome.

During the last visit (5 years of age), she remains clinically stable without progressive skeletal deformity or additional neurological complications. She attends mainstream education with individualized educational support.

### 3.2. Genetic Testing

The age at genetic testing ranged from 3 to 16 years (mean age: 8.7 years; median age: 8 years). Genetic variants in *RPS6KA3* were identified in all patients, with three variants classified as pathogenic, three as likely pathogenic, and four as variants of uncertain significance (Table 2—*RPS6KA3* variants in patients with Coffin–Lowry syndrome at CRGMDj) before assessing segregation. Missense variants were most frequent (*n* = 6); two patients carried intronic mutations; there was one frameshift mutation and one nonsense variant.

All variants were *de novo*. Adding the PM6 criteria to the variants of the case series changes the pathogenicity class upwards for most variants (*), with (**) being reclassified from VUS to likely pathogenic. Supplementary Materials Table S2 details *in silico* scores for these variants (CADD, REVEL, SIFT) and gnomAD (aggregated) frequency. ChrX(GRCh37):g.20196049_20196050insN[6095]; NM_004586.3:c.846–848_846–847insN[6095]; *RPS6KA3* r.845–846ins(846–877_846–848;n [98]); p.(Ala283Phefs18*) is the insertion of a transposable element LINE-1 in intron 10 of the *RPS6KA3* gene, constituted of 6081 bp LINE1 sequence and 14 bp of a duplication of the target site at the genomic DNA level. At the RNA level, there is the retention of a fragment of 30 bp intron 10 and 98 bp of the LINE-1 sequence, which probably leads to a premature strop codon, explaining the lack of residual functional protein.

Supplementary Materials Table S1 captures genotype–phenotype correlates—intellectual disability and skeletal severity; presence of stimulus induces drop episodes (SIDEs) for each variant. Of note, the degree of intellectual disability was ranked “severe” only for cases 3 and 4, case 6 showed an advanced degree of kyphoscoliosis, whereas case 9 showed no primal involvement, and only case 7 showed signs of SIDEs. Cardiac evaluation did not find any cardiac anomalies in this case series.

Diagnostic delay (as shown in Supplementary Materials Table S1) had a wide range—between 2 and 16 years from the onset of first symptoms, with a median of 7 years. The two female cases we have included had a diagnostic delay of 2 and 9 years, respectively.

## 4. Discussion

The present study provides a detailed overview of the diagnostic challenges associated with CLS, based on a single-center case series. Our cohort shows both areas of overlap with previously reported case series and notable differences that merit discussion.

Consistent with earlier studies, we observed a similar range of core clinical features, including global developmental delay, hypotonia, and characteristic facial morphology and a predominance of truncating *RPS6KA3* variants [1,31]. However, our data expand the phenotypic spectrum by documenting several less commonly reported manifestations, accompanied by a novel genetic variant captured by contemporary sequencing approaches.

We highlight several gaps in the early recognition of CLS, reflected in the substantial delay between symptom onset and clinical suspicion. This diagnostic latency may likely be caused by the overlap of early CLS manifestations—global developmental delay, hypotonia, and behavioral dysregulation—with common neurodevelopmental disorders. Similar delays have been reported in previous studies, underscoring the difficulty of identifying CLS in the absence of clear dysmorphic features or a known family history [13,20,33]. Regional differences in genetic testing availability or accessibility contributed to longer delays in some cases as well [16,19,20].

Together, this shows consistency over key clinical patterns and the added value of extending the phenotypic and molecular landscape of Coffin–Lowry syndrome with our findings.

### 4.1. Challenges in Diagnosing CLS

Overall, our findings emphasize that, although diagnostic technologies have advanced significantly, clinical recognition of CLS remains challenging. The combination of subtle early symptoms, phenotypic variability, and rare genetic etiology continues to contribute to delayed diagnosis and unstandardized case management practices. The variability in female presentations complicates diagnosis and genetic counseling, as you can see from the clinical vignette presented. Additionally, long-term outcome studies are limited, making prognostic counseling challenging.

CLS is characterized by a high degree of clinical variability, with significant sex-related differences in phenotypic expression [16,23,34]. Both females and (occasionally) males can exhibit mild ID and subtle dysmorphic features, with a risk of remaining unrecognized or being misdiagnosed as a different neurodevelopmental disorder.

In addition, the diagnosis of CLS can be further complicated by overlapping clinical features with other syndromic forms of intellectual disability, which may contribute to misdiagnosis or delayed recognition [5].

Across the phenotypic spectrum of *RPS6KA3*-related intellectual disability, varying degrees of clinical involvement can be documented, with classical CLS at the more severe end. X-linked intellectual developmental disorder-19 (XLID19, OMIM #300844), a phenotype at the milder end, is characterized by less severe impaired intellectual development due to genetic variants resulting in residual protein function [31].

Disorders that closely resemble CLS and should be considered in the differential diagnosis include Alpha-Thalassemia X-Linked Intellectual Disability Syndrome (ATR-X; OMIM #301040), FG syndrome type 1 (OMIM #305450), Borjeson–Forssman–Lehmann syndrome (BFLS; OMIM #301900), Williams syndrome (OMIM #194050), and Pitt–Hopkins syndrome (OMIM #610954) [2,3].

Approximately 70–80% of probands have no family history of CLS, representing sporadic cases, while 20–30% have additional affected family members [13]. None of the 10 index cases had a positive family history. The syndrome shows no apparent clustering by geographic region or ethnic background, in keeping with its genetic basis.

In our cohort, the age at onset is similar to previously reported cases [1,13]; nevertheless, 7 out of 10 patients received a definitive diagnosis more than 4 years (Supplementary Materials Table S1) following the onset of symptoms. There is insufficient literature evidence to compute exact onset-to-diagnosis intervals, because case reports/case series rarely report both the precise age of the first symptom and the age/date of definitive genetic diagnosis in the same patient [28]. This finding, we believe, highlights critical gaps in medical healthcare accessibility and early recognition and testing availability in several geographic areas in an already challenging disease presentation. We find that reports should clearly state first symptom onset/clinical concern age at referral for genetic testing/age at definitive molecular diagnosis to permit the calculation of diagnostic delay and stratified analyses by sex, severity, and geography to capture these gaps.

The rarity of CLS and limited clinician familiarity with its early phenotypic spectrum further contribute to diagnostic delays. Many individuals consult multiple healthcare professionals and are often misdiagnosed at least once before receiving the correct diagnosis. A contributing factor to these delays is represented by the limited access to centers of expertise and healthcare professionals with experience in rare diseases [1,13]. This underscores the importance of increasing awareness of CLS among pediatric and genetic specialists, as well as the potential utility of the earlier implementation of comprehensive genomic testing in patients with undiagnosed developmental disorders.

Establishing a diagnosis relies heavily on molecular testing, as there are no specific laboratory findings to support a clinical diagnosis [7]. Next-generation sequencing and high-resolution genomic arrays can detect frequent types of variants, as well as small intragenic duplications and other complex mutations [35,36]. Methylation studies can assess X-chromosome inactivation patterns in females, a process that can influence phenotypic variability [37].

Access to timely and comprehensive genetic testing remains uneven, particularly in regions where sequencing is not routinely reimbursed or where copy-number analysis requires a referral to specialized centers. Access to molecular testing may also be delayed by limited cost-related barriers or extended processing times, which further postpone etiologic confirmation.

### 4.2. Genetic Variation and Genetic Testing in CLS

Over 75 distinct pathogenic variants have been identified in 250 unrelated CLS patients, with mutations distributed throughout the gene [1]. The spectrum includes missense mutations, nonsense variants, splicing errors, and short deletion or insertion events [1,16,19,31,38]. Approximately 57% of variants result in premature translation termination, with the vast majority predicted to cause loss of function [5]. About two-thirds of cases arise from *de novo* mutations [1]; in our case series, all variants were *de novo*.

We report 10 variants in the *RPS6KA3* gene: six missense mutations, two nonsense variants, and two variants in non-coding or intronic regions, including one involving a LINE-1 transposable element insertion in intron 10. These alterations were distributed throughout the gene, and several are consistent with the known loss-of-function disease mechanism in CLS [39]. A recently published systematic review [40] did not report any of the variants we have described in the current article. It may be that the RPS6KA3 single-nucleotide or small indel variant are not reported explicitly with a precise HGVS nomenclature; many additional variants described in cohort studies, mutation catalogs, and older reviews are cited as counts or summarized, but their individual c.HGVS calls are not present in the provided excerpts. This underlines the need for structured reporting and the use of standardized nomenclature.

The splice-site variant c.1765–2A>G was identified at the acceptor site of intron 18. A similar mutation affecting the donor site of the same intron (c.1765+1G>A) was previously reported by Hanauer et al. (2002) [1] and was shown to cause the skipping of exon 18. Splice-site mutations in *RPS6KA3* are a known mechanism in Coffin–Lowry syndrome, resulting in exon skipping and truncated protein products. Exon skipping has been estimated to account for approximately 7% of pathogenic mutations in *RPS6KA3* [5].

The frameshift variant c.1740delC, predicted to lead to a downstream premature stop codon (p.Tyr580Ter), was detected in case no. 3. This type of loss-of-function variant is consistent with the known pathogenic mechanisms of *RPS6KA3* in CLS, particularly when affecting the C-terminal kinase domain [7].

Six missense variants were detected, c.578A>T, c.1696A>G, c.791G>A, c.1351A>C, c.1825T>G, and c.10G>C, with four of them initially classified as VUS before considering segregation (Table 2). All of these occur at conserved residues within or near the functional kinase domains of RSK2. Although some of them have not been previously reported in the literature or major databases, the domain context, conservation of affected residues and missense predictors suggest possible deleterious effects. Variants of uncertain significance are becoming increasingly reported, and their interpretation has proven to be complex. As a consequence, clinicians face difficulties in the counseling and management of these cases [14]. Variant reclassification may occur: (1) As our case series shows, segregation is crucial, as it may change ACMG classification and must be evaluated in order to ensure as complete an evaluation as possible. (2) New cases may be reported, and their comprehensive description may help complete our understanding of CLS. (3) Functional validation may change our understanding of variant impact; however, tools are lacking, and assays to directly address this are not available or routine in most laboratories.

The nonsense variant c.334C>T (p.Arg112Ter), identified in case no. 8, introduces a premature stop codon within the N-terminal kinase domain, likely resulting in a truncated and non-functional RSK2 protein.

One of the reported variants consists of a large insertion (6095 bp) of unknown nucleotides, likely representing a mobile element insertion (LINE-1, Long Interspersed Nuclear Element-1), which may disrupt the reading frame or regulatory sequences of the *RPS6KA3* gene. A similar LINE-1 insertion has previously been reported in intron 3, leading to exon 4 skipping and subsequently to a premature stop codon and a truncated protein; LINE-1 insertion may be a possible pathogenic mechanism for CLS [1]. Although the phenotype of this patient was highly suggestive, genetic confirmation could not be achieved using DNA-based sequencing technologies available at that time. A definitive molecular diagnosis was ultimately obtained using RNA sequencing, trio-genome sequencing and long-range PCR which revealed the presence of a LINE-1 transposable element insertion, similarly to others [39]. Segregation analysis demonstrated that the genetic variant was *de novo*.

With the advancement of NGS technologies that are becoming more accessible and used in clinical practice, the identification of novel genetic variants has improved diagnostic accuracy and modestly contributed genotype–phenotype correlations [41]. Genotype–phenotype correlations remain poorly understood, with no consistent relationship between mutation type and clinical severity [1,13]. In line with prior genotype–phenotype studies [15,16,18,19,31,33,35], truncating variants seem to be associated with more severe neurodevelopmental impairment, whereas missense changes tended to yield a broader range of clinical presentations and sometimes a milder range of clinical features. Our reported variants align with this pattern, showing a phenotypic severity comparable to previously documented truncating or loss-of-function alleles. However, some individuals exhibited milder or atypical features, underscoring the incomplete penetrance and variable expressivity that have been repeatedly emphasized in the CLS literature. Overall, we recognize that the sample size in our case series is relatively small, reflecting the rarity of Coffin–Lowry syndrome, and limiting the statistical power to fully explore subtle genotype–phenotype correlations.

In our study, genetic diagnoses were established exclusively through sequencing-based methods (clinical exome or targeted gene sequencing). As a result, large deletions or duplications involving *RPS6KA3* may not have been detected, and this is one of the limitations of our study. Larger-scale or exon-level events (duplications, in-frame exon deletions, and mobile element insertion) have been described less frequently in CLS [40], with one larger study placing CNV distribution at around 3% of cases [5]. Most CNV observations are isolated families or small series [22,42]. Arguably, this larger rearrangement may have been missed and consequently misrepresented. Therefore MLPA, array-CGH, targeted CGH, or protein/immunoblot assays are required to detect deletions/duplications in mutation-negative, clinically suggestive cases [5,40]

### 4.3. Implications for Management of CLS

Management of CLS needs to take into account multisystemic involvement and diverse clinical manifestations. CLS patients require multidisciplinary team care involving neurology, orthopedics, cardiology, pulmonology, and clinical genetics specialists, as well as psychological and behavioral therapy.

Multidisciplinary evaluation often requires repeated appointments and fragmented care, with specialized expertise available in only a few centers. Long-term follow-up planning is further hindered by variability in local resources and the absence of standardized follow-up guidelines. Additionally, families frequently required repeated counseling regarding prognosis, recurrence risk, and the management of behavioral and neurodevelopmental difficulties, yet genetic counseling services were consistently only available at reference centers.

The multisystem involvement documented across our cohort reinforces the need for comprehensive assessments. Cardiovascular, audiological, skeletal, and neurological abnormalities were observed in patterns consistent with the earlier literature, emphasizing the value of multidisciplinary care and periodic surveillance [12,24,25,27,29].

The current study contributes to the expanding mutational spectrum of *RPS6KA3* by identifying several pathogenic and likely pathogenic variants. The retrospective design introduces variability in the completeness and quality of the clinical information available, and some phenotypic features may have been underreported in medical records. Longitudinal data were incomplete for several individuals, which limited our capacity to systematically assess disease progression, long-term management outcomes, and evolving neurodevelopmental profiles. These limitations should be considered when interpreting the results, and they highlight the need for larger, prospective, multicenter studies. However, our experience supports published recommendations that individuals with CLS should undergo regular evaluations by cardiology, audiology, neurology, and clinical genetics teams to optimize both diagnostic accuracy and long-term management.

#### 4.3.1. Neurodevelopmental and Cognitive Management

CLS patients experience variable degrees of intellectual disability and behavioral challenges that require specialized therapeutic and educational interventions to reach their developmental potential. In our case series, it was these symptoms that mostly directed the cases to our expertise center and resulted in their genetic diagnosis. Management strategies involve the initial assessment of symptoms and surveillance of their development, including motor, adaptive, cognitive, and speech-language evaluations, with referral to individualized therapeutic programs tailored to each patient’s specific needs [43].

Behavioral concerns, including sleep disturbances, attention-deficit/hyperactivity disorder (ADHD), features suggestive of autism spectrum disorder (ASD), as well as aggressivity, anxiety, and other psychiatric comorbidities, should be addressed using standard clinical guidelines, including pharmacologic management when appropriate [3].

Access to physical, occupational, behavioral, and speech therapies may be limited, delaying functional progress and quality-of-life improvements.

#### 4.3.2. Seizures and Stimulus-Induced Drop Episodes (SIDEs)

Neurological complications such as stimulus-induced drop episodes (SIDEs), characterized by sudden, non-epileptic atonic collapses triggered by tactile or auditory stimuli, occur in approximately 10–20% of patients and are often refractory to standard anticonvulsant therapy, necessitating tailored pharmacologic approaches and supportive measures. In our case series, they were reported in only one case. SIDEs may present variable patterns over time; perhaps, for this reason, long-term control remains limited despite the use of various therapeutic agents—including benzodiazepines, antiseizure medications (ASMs), selective serotonin reuptake inhibitors (SSRIs), and tricyclic antidepressants [1,3,7,17].

In addition to SIDEs, individuals with CLS may present a range of neurological manifestations, including epileptic seizures, congenital hypotonia, brain abnormalities, and sensorineural hearing deficits [2,3]. Accurate differentiation between epileptic seizures and SIDEs is essential, as seizures often present with focal onset, tonic–clonic movements of a longer duration that require neurological expertise and standard antiepileptic treatment [7].

#### 4.3.3. Skeletal and Growth Abnormalities

Musculoskeletal manifestations include progressive kyphoscoliosis, a short stature with disproportionately short lower limbs, tapered fingers, pectus carinatum or excavatum, and calcifications or hypertrophy of ligamentum flavum, with progressive deterioration observed over time in the affected individual, as seen in our case series as well as in the literature [3,36,37]. A total of 80% of CLS patients have a spinal abnormality that influences the quality of life, and severe deformities can lead to cardiorespiratory complications and significant neurological compromise. In these situations, earlier and more aggressive surgical management is strongly recommended [29,37]. Patients with CLS are usually uncooperative and have multiple cranio-facial dysmorphisms, making classical bag-mask ventilation and orotracheal intubation less feasible or impossible to achieve [41].

In addition to surgical intervention, individuals with CLS require periodic evaluation by orthopedics, physical medicine, and rehabilitation specialists. Regular assessment of mobility, daily activities and the need for adaptive devices is essential for gross and fine motor skills improvement and functional independence [3,14].

Growth abnormalities necessitate the close monitoring of growth parameters, including height, weight, and head circumference, along with nutritional status [14]. Although short stature is frequently observed, current evidence does not support the use of growth hormone therapy in CLS [22]. To prevent obesity, regular nutritional assessment and height–weight ratio monitoring are essential.

#### 4.3.4. Cardiac and Respiratory Issues

In our cohort, none of the cases showed cardiac anomalies. CLS patients may present significant cardiac and respiratory involvement, particularly valvular abnormalities, various forms of cardiomyopathy, and obstructive sleep apnea, all of which may contribute to premature mortality. Cardiac evaluation, including electrocardiography and echocardiography, is recommended at diagnosis and periodically. Respiratory concerns include obstructive sleep apnea and restrictive lung disease secondary to kyphoscoliosis, requiring evaluation and intervention by specialized care teams [3,25].

#### 4.3.5. Multidisciplinary Care Approach

CLS is a complex and rare genetic disease with no specific or curative treatment. A multidisciplinary care approach is important for improving quality of life and reducing possible complications. This can only be achieved through the collaboration of pediatrics, neurology, genetics, family medicine, sleep medicine, orthopedics, cardiology, ENT, stomatology, surgery, and anesthesiology specialists. CLS patients need specific therapies for behavioral and developmental challenges, hearing aid, complementary measures for preventing falls, correction of spine deformities, treatment for sleep apnea, and continuous medication to prevent seizures.

Our findings highlight several diagnostic and management challenges that remain under-recognized in Coffin–Lowry syndrome (CLS), particularly regarding delayed clinical suspicion, variability in neurodevelopmental trajectories, and the heterogeneous expression of associated systemic anomalies. The marked discrepancy between age at symptom onset and age at clinical suspicion underscores persistent gaps in early recognition, even in the presence of characteristic phenotypic features. This pattern is consistent with previous reports indicating that early developmental delays and behavioral dysregulation in CLS frequently overlap with more common neurodevelopmental disorders, resulting in diagnostic latency. Our data also reinforce the clinical value of multidisciplinary assessments, as multi-system involvement was evident in nearly all patients, highlighting the importance of comprehensive cardiovascular, audiological, and neurological surveillance.

## 5. Conclusions

Coffin–Lowry syndrome remains a diagnostically challenging condition due to its heterogeneous clinical presentation and overlap with more common neurodevelopmental disorders. Our single-center experience demonstrates that delayed recognition persists even when characteristic features eventually emerge. The newly identified *RPS6KA3* variants reported here expand the known mutational landscape and illustrate the ongoing need for comprehensive molecular and clinical characterization to refine genotype–phenotype correlations. Early diagnosis of CLS is necessary for adequate management, providing important improvements in quality of life and the development of patients. Our findings underline the broad phenotypic variability, the challenges encountered by inconsistent manifestations in females, and the frequent delay in establishing a genetic diagnosis. Management is complex and intertwined with multiple specialties. This cohort adds to the limited data available on CLS.

Improved diagnostic pathways, routine multidisciplinary follow-up, and the systematic incorporation of new variants into curated databases will be essential to enhancing early recognition, standardizing care, and advancing our understanding of the diverse clinical trajectories associated with CLS. Further longitudinal studies are needed to better define the natural history of the disorder and to support the development of evidence-based management recommendations.

## Figures and Tables

**Table 1:** Clinical features observed in patients with Coffin–Lowry syndrome (*n* = 10) at CRGMDj.

Clinical Feature	No. of Patients (*n*)	Frequency	Age of Onset	Severity
Male	8	80%	–	–
Developmental delay/ intellectual disability	10	100%	3–18 months	Moderate to severe
Craniofacial dysmorphism	10	100%	Infancy	Mild to moderate
Prominent forehead	10	100%	Infancy	Mild
Down-slanting palpebral fissures	10	100%	Infancy	Mild
Broad nasal bridge	10	100%	Infancy	Mild
Thick lips/everted lower lip	10	100%	Infancy	Mild
Skeletal abnormalities	9	90%	3–8 years	Moderate
Kyphosis/scoliosis	6	60%	5–12 years	Moderate to severe
Pectus excavatum	4	40%	3–8 years	Mild to moderate
Hyperextensible joints	5	50%	2–6 years	Mild
Hypotonia	7	70%	Neonatal/early infancy	Mild to moderate
Drop episodes/atonic seizures	1	10%	3 years	Moderate
Hearing loss (sensorineural)	5	50%	2–6 years	Mild to moderate
Speech delay	10	100%	2–3 years	Moderate to severe
Behavioral disturbances	6	60%	4–10 years	Mild to moderate
Anxiety/aggressivity	4	40%	5–10 years	Mild to moderate
Autistic features	2	20%	3–6 years	Mild
Cardiac anomalies (e.g., valve defects)	0	–	–	–
Delayed diagnosis (>2 years)	7	70%	–	–

**Table 2:** *RPS6KA3* variants in patients with Coffin–Lowry syndrome at CRGMDj.

Case No	Sex	Age at Diagnosis (Years)	*RPS6KA3* Variant (NM_004586.3)	Region	Variant Type	Protein Change	Pathogenicity ACMG Criteria	
1	Male	6	c.1765-2A>G	Intron 18	Non-coding		Pathogenic *	PVS1, PM2, PM6
2	Female	3	c.578A>T	Exon 7	Missense	p.Asp193Val	Likely Pathogenic	PM1, PP2, PM2, PM6
3	Male	4	c.1740delC	Exon 18	Frameshift indel	p.Tyr580Ter	Pathogenic	PVS1, PM2, PP5, PM6
4	Male	12	c.1696A>G	Exon 18	Missense	p.Lys566Glu	Likely Pathogenic **	PP3, PM1, PM2, PM6
5	Male	9	c.846–848_846–847insN [6095]	Intron 10	Insertion of a transposable element LINE-1		Pathogenic	PVS1, PM4, PM6
6	Male	16	c.791G>A	Exon 10	Missense	p.Gly264Asp	Likely pathogenic **	PM1, PP3, PM2, PM6
7	Male	14	c.1351A>C	Exon 15	Missense	p.Lys451Gln	Likely Pathogenic	PP3, PP2 PM2, PM6
8	Male	7	c.334C>T	Exon 5	Nonsense	p.Arg112Ter	Pathogenic	PVS1, PP5, PM2
9	Female	11	c.1825T>G	Exon 19	Missense	p.Tyr609Asp	Likely Pathogenic **	PP3, PM6 PM1, PM2, PM6
10	Male	5	c.10G>C	Exon 1	Missense	p.Ala4Pro	Likely Pathogenic **	PM2, PP2, PM6

## Data Availability

The raw data supporting the conclusions of this article will be made available by the authors on request.

## Supplementary Materials

The following supporting information can be downloaded at: https://www.mdpi.com/article/10.3390/diagnostics16070990/s1, Table S1. Genotype–phenotype correlation in patients with Coffin–Lowry syndrome at CRGMDj; Table S2. RPS6KA3 variants *in silico* scores.

## References

[1] Hanauer A., Young I.D. (2002). Coffin-Lowry syndrome: Clinical and molecular features. J. Med. Genet..

[2] Pereira P.M., Schneider A., Pannetier S., Heron D., Hanauer A. (2010). Coffin-Lowry syndrome. Eur. J. Hum. Genet..

[3] Rogers R.C., Abidi F.E., Adam M.P., Bick S., Mirzaa G.M., Pagon R.A., Wallace S.E., Amemiya A. (1993). RPS6KA3-Related Intellectual Disability. GeneReviews^®^.

[4] Delaunoy J., Abidi F., Zeniou M., Jacquot S., Merienne K., Pannetier S., Schmitt M., Schwartz C., Hanauer A. (2001). Mutations in the X-linked RSK2 gene (RPS6KA3) in patients with Coffin-Lowry syndrome. Hum. Mutat..

[5] Delaunoy J.P., Dubos A., Marques Pereira P., Hanauer A. (2006). Identification of novel mutations in the RSK2 gene (RPS6KA3) in patients with Coffin-Lowry syndrome. Clin. Genet..

[6] Schneider A., Maas S.M., Hennekam R.C., Hanauer A. (2013). Identification of the first deep intronic mutation in the RPS6KA3 gene in a patient with a severe form of Coffin-Lowry syndrome. Eur. J. Med. Genet..

[7] Jacquot S., Zeniou M., Touraine R., Hanauer A. (2002). X-linked Coffin-Lowry syndrome (CLS, MIM 303600, RPS6KA3 gene, protein product known under various names: Pp90(rsk2), RSK2, ISPK, MAPKAP1). Eur. J. Hum. Genet..

[8] Gonzalez L., Sebrie C., Laroche S., Vaillend C., Poirier R. (2023). Delayed postnatal brain development and ontogenesis of behavior and cognition in a mouse model of intellectual disability. Neurobiol. Dis..

[9] Lizcano-Perret B., Vertommen D., Herinckx G., Calabrese V., Gatto L., Roux P.P., Michiels T. (2024). Identification of RSK substrates using an analog-sensitive kinase approach. J. Biol. Chem..

[10] Spirrison A.N., Lannigan D.A. (2024). RSK1 and RSK2 as therapeutic targets: An up-to-date snapshot of emerging data. Expert Opin. Ther. Targets.

[11] Lv Y., Zhu L., Zheng J., Wu D., Shao J. (2018). Growth Concerns in Coffin-Lowry Syndrome: A Case Report and Literature Review. Front. Pediatr..

[12] Pantani A., Rizzi S., Spagnoli C., Cesaroni C.A., Cavalli A., Caraffi S.G., Peruzzi A., Peluso F., Russo R., Scala I. (2025). Myoclonic reflex and non-reflex seizures in a female child with Coffin-Lowry syndrome: Clinical vignette. Epileptic Disord..

[13] Tise C.G., Matalon D.R., Manning M.A., Byers H.M., Grover M. (2022). Short Bones, Renal Stones, and Diagnostic Moans: Hypercalcemia in a Girl Found to Have Coffin-Lowry Syndrome. J. Investig. Med. High Impact Case Rep..

[14] Carey J.C., Cassidy S.B., Battaglia A., Viskochil D. (2021). Cassidy and Allanson’s Management of Genetic Syndromes.

[15] Sayar E., Gokce Altas G., Sezer A., Kolkiran A., Ucan B., Olgac A. (2026). A 2-year-old girl with merged phenotypes: Galactosemia and Coffin-Lowry syndrome. J. Pediatr. Endocrinol. Metab..

[16] Cong Y., Jin H., Wu K., Wang H., Wang D. (2022). Case Report: Chinese female patients with a heterozygous pathogenic RPS6KA3 gene variant c.898C>T and distal 22q11.2 microdeletion. Front. Genet..

[17] Hahn J.S., Hanauer A. (2012). Stimulus-induced drop episodes in Coffin-Lowry syndrome. Eur. J. Med. Genet..

[18] Gursoy S., Hazan F., Cetinoglu E. (2022). Novel RPS6KA3 mutations cause Coffin-Lowry syndrome in two patients and concurrent compulsive eyebrow-pulling behavior in one of them. Psychiatr. Genet..

[19] Song A., Im M., Kim M.S., Noh E.S., Kim C., Jang J., Lee S.M., Ki C.S., Cho S.Y., Jin D.K. (2023). First female Korean child with Coffin-Lowry syndrome: A novel variant in RPS6KA3 diagnosed by exome sequencing and a literature review. Ann. Pediatr. Endocrinol. Metab..

[20] Tan S.L., Ahmad Narihan M.G.B., Koa A.J. (2023). An unexpected presentation of very severe hypertriglyceridemia in a boy with Coffin-Lowry syndrome: A case report. BMC Pediatr..

[21] Spodzieja K., Olczak-Kowalczyk D. (2022). Premature Loss of Deciduous Teeth as a Symptom of Systemic Disease: A Narrative Literature Review. Int. J. Environ. Res. Public Health.

[22] Castelluccio V.J., Vetrini F., Lynnes T., Jones J., Holloway L., Belonis A., Breman A.M., Graham B.H., Sapp K., Wilson T. (2019). An unusual cause for Coffin-Lowry syndrome: Three brothers with a novel microduplication in RPS6KA3. Am. J. Med. Genet. Part A.

[23] Labonne J.D., Chung M.J., Jones J.R., Anand P., Wenzel W., Iacoboni D., Layman L.C., Kim H.G. (2016). Concomitant partial exon skipping by a unique missense mutation of RPS6KA3 causes Coffin-Lowry syndrome. Gene.

[24] Ali T., Albarni A., Guez M., Akerstedt J. (2025). Cardiovascular Collapse During Scoliosis Surgery in a Patient with Coffin-Lowry Syndrome and Mesocardia. Cureus.

[25] Ghose S., Nisar F., Aleem B.A. (2024). Airway management of a patient with Coffin-Lowry syndrome: A case report. BMC Anesthesiol..

[26] Kim J.S., Ilaria S., Hundal J., Bhise V. (2025). Coffin-Lowry Syndrome: A Case of Clinical Convergence for Psychology, Neuropsychology, Psychiatry, Genetics, and Neurology. J. Child. Neurol..

[27] Wakami T., Yoshizawa K., Maeda T., Mori O., Tamura N. (2022). Mitral valve repair and tricuspid annuloplasty for Coffin-Lowry syndrome. Asian Cardiovasc. Thorac. Ann..

[28] Hunter A.G. (2002). Coffin-Lowry syndrome: A 20-year follow-up and review of long-term outcomes. Am. J. Med. Genet..

[29] Welborn M., Farrell S., Knott P., Mayekar E., Mardjetko S. (2018). The natural history of spinal deformity in patients with Coffin-Lowry syndrome. J. Child. Orthop..

[30] Soyler A.K., Serel Arslan S., Demir N., Karaduman A.A., Duger T. (2024). Chewing and swallowing training in Coffin-Lowry syndrome: A case report. J. Oral Rehabil..

[31] Di Stazio M., Bigoni S., Iuso N., Vuch J., Selvatici R., Ulivi S., d’Adamo P.A. (2021). Identification of a New Mutation in RSK2, the Gene for Coffin-Lowry Syndrome (CLS), in Two Related Patients with Mild and Atypical Phenotypes. Brain Sci..

[32] Richards S., Aziz N., Bale S., Bick D., Das S., Gastier-Foster J., Grody W.W., Hegde M., Lyon E., Spector E. (2015). Standards and guidelines for the interpretation of sequence variants: A joint consensus recommendation of the American College of Medical Genetics and Genomics and the Association for Molecular Pathology. Genet. Med..

[33] Touma Boulos M., Moukarzel A., Yammine T., Salem N., Souaid M., Farra C. (2021). Novel missense mutation c.1784A>G, p.Tyr595Cys in RPS6KA3 gene responsible for Coffin-Lowry syndrome in a family with variable features and diabetes 2. Clin. Dysmorphol..

[34] Martinez-Garay I., Ballesta M.J., Oltra S., Orellana C., Palomeque A., Molto M.D., Prieto F., Martinez F. (2003). Intronic L1 insertion and F268S, novel mutations in RPS6KA3 (RSK2) causing Coffin-Lowry syndrome. Clin. Genet..

[35] Field M., Tarpey P., Boyle J., Edkins S., Goodship J., Luo Y., Moon J., Teague J., Stratton M.R., Futreal P.A. (2006). Mutations in the RSK2(RPS6KA3) gene cause Coffin-Lowry syndrome and nonsyndromic X-linked mental retardation. Clin. Genet..

[36] Phillips C., Parkinson A., Namsrai T., Chalmers A., Dews C., Gregory D., Kelly E., Lowe C., Desborough J. (2024). Time to diagnosis for a rare disease: Managing medical uncertainty. A qualitative study. Orphanet J. Rare Dis..

[37] Faye F., Crocione C., Anido de Pena R., Bellagambi S., Escati Penaloza L., Hunter A., Jensen L., Oosterwijk C., Schoeters E., de Vicente D. (2024). Time to diagnosis and determinants of diagnostic delays of people living with a rare disease: Results of a Rare Barometer retrospective patient survey. Eur. J. Hum. Genet..

[38] Jin H., Li H., Qiang S. (2022). Coffin-Lowry Syndrome Induced by RPS6KA3 Gene Variation in China: A Case Report in Twins. Medicina.

[39] Biehler M., Tarabeux J., Calmels N., Feger C., Piton A., Muller J., Schluth-Bolard C., Streata I., Montano C., Gerard B. LINE-1 retrotransposon insertion in RPS6KA3 as a cause of Coffin-Lowry syndrome. Proceedings of the 56th Annual Conference of the European-Society-of-Human-Genetics (ESHG).

[40] Maity S., Montion M., Boothe D., Attarpour M., Mageto I., Agarwal S., Patel R., Lark C., Cardona Valentin N., Quinones-Budel E. (2025). Coffin-Lowry syndrome: A systematic review of RPS6KA3 confirmed cases and implications for diagnosis and counseling. Front. Genet..

[41] Miyata Y., Saida K., Kumada S., Miyake N., Mashimo H., Nishida Y., Shirai I., Kurihara E., Nakata Y., Matsumoto N. (2018). Periventricular small cystic lesions in a patient with Coffin-Lowry syndrome who exhibited a novel mutation in the RPS6KA3 gene. Brain Dev..

[42] Marques Pereira P., Heron D., Hanauer A. (2007). The first large duplication of the RSK2 gene identified in a Coffin-Lowry syndrome patient. Hum. Genet..

[43] Rogers R.C., John C., Carey A.B., Viskochil D., Suzanne B.C. (2020). C12. Coffin–Lowry Syndrome. Cassidy and Allanson’s Management of Genetic Syndromes.

