# Supplementary material for: Challenges in Diagnosis and Management of Coffin–Lowry Syndrome—Single-Center Experience
Source: Diagnostics (Basel). 2026 Mar 25;16(7):990. doi: 10.3390/diagnostics16070990 (PMC13073513; doi:10.3390/diagnostics16070990)
Supplement: Supplementary file 1 [file diagnostics-16-00990-s001.zip › diagnostics-4155363-supplementary.pdf]

Supplementary Table S1. Genotype –phenotype correlation in patients with Coffin–Lowry syndrome at CRGMDj.

Case no	Sex	Age at diagnosis (years)	*RPS6KA3* variant (NM_004586.3)	Diagnostic delay since first reported symptoms/signs (years)	Intellectual disability (DSM-5)	Skeletal severity scale (spinal deformity)	Stimulus-induced drop episodes
1	Male	6	c.1765-2A>G	2	Moderate	Moderate	No
2	Female	3	c.578A>T	2	Moderate	Moderate	No
3	Male	4	c.1740delC	2	Severe	Moderate	No
4	Male	12	c.1696A>G	10	Severe	Moderate	No
5	Male	9	c.846-848_846-847insN[6095]	7	Moderate	Moderate	No
6	Male	16	c.791G>A	16	Moderate	Severe	No
7	Male	14	c.1351A>C	14	Moderate	Moderate	Moderate
8	Male	7	c.334C>T	7	Moderate	Severe	No
9	Female	11	c.1825T>G	9	Moderate	No skeletal signs	No
10	Male	5	c.10G>C	4	Moderate	Moderate	No

Supplementary Table S2. *RPS6KA3* variants in silico scores.

RPS6KA3 variant (NM_004586.3)	CADD	REVEL	PolyPhen-2	SIFT	gnomAD (aggregated) frequency
c.1765-2A>G	34	N/A	N/A	N/A	0 Alleles of 113,096<br>0 Homozygote<br>N/A Individuals
c.578A>T	29.6	Deleterious (Strong) (0.98)	N/A	Deleterious (Supporting) (0)	0 Alleles of 1,155,803<br>0 Homozygote<br>N/A Individuals
c.1740delC	34	N/A	N/A	N/A	0 Alleles of 1,209,724<br>0 Homozygote

					N/A Individuals
c.1696A>G	34	Deleterious (Supporting) (0.65)	N/A	Uncertain (0.001)	0 Alleles of 1,206,396 0 Homozygote N/A Individuals
c.846-848_846-847insN[6095]					
c.791G>A	29.6	Deleterious (Supporting) (0.76)	N/A	Uncertain (0.001)	0 Alleles of 1,181,235 0 Homozygote N/A Individuals
c.1351A>C	29.8	Deleterious (Moderate) (0.92)	N/A	Uncertain (0.001)	0 Alleles of 1,191,921 0 Homozygote N/A Individuals
c.334C>T	39	N/A	N/A	N/A	0 Alleles of 1,154,409 0 Homozygote N/A Individuals
c.1825T>G	29.4	Deleterious (Supporting) (0.76)	N/A	Deleterious (Supporting) (0)	0 Alleles of 1,182,263 0 Homozygote N/A Individuals
c.10G>C	25.1	Uncertain (0.33)	N/A	Uncertain (0.003)	0 Alleles of 1,144,474 0 Homozygote N/A Individuals
